# *In vivo* Study of Chalcone Loaded Carbon Dots for Enhancement of Anticancer and Bioimaging Potencies

**DOI:** 10.7150/ntno.80030

**Published:** 2023-03-12

**Authors:** Mochamad Zakki Fahmi, Yu-Yu Aung, Musbahu Adam Ahmad, Alfinda Novi Kristanti, Satya Candra Wibawa Sakti, Oka Pradipta Arjasa, Hwei Voon Lee

**Affiliations:** 1 Department of Chemistry, Airlangga University, Surabaya 60115, Indonesia.; 2 Supramodification Nano-micro Engineering (SPANENG) Research Group, Airlangga University, Surabaya 60115, Indonesia.; 3 Advanced Materials Research Centre - National Research and Innovation Agency, Central Jakarta 10340, Indonesia.; 4 Nanotechnology & Catalysis Research Centre (NANOCAT), Level 3, Block A, Institute for Advanced Studies, Universiti Malaya, Kuala Lumpur 50603, Malaysia.

**Keywords:** Carbon dots, chalcone, drug release, *in vitro* and *in vivo* assay

## Abstract

The fluorescent imaging and drug delivery utilizing carbon dots nanomaterials (CDs) have attracted tremendously due to their unique optical ability and outstanding biocompatibility. Herein, we reported a new design of chalcone-loaded carbon dots (Chalcone-APBA-CDs) to serve chalcone transport onto cancer cells and enhance the CDs bioimaging and antitumor activity. The boronic acid was directly introduced to carbon dots (CDs) via pyrolysis process to drive CDs specifically to the cancer cell, and chalcone was mediated on CDs by ultrasonication to perform facile release of the drug delivery model. The successfully synthesized Chalcone-APBA-CDs were proved by their chemical structure, fluorescent activities, *in vitro* and *in vivo* analyses, and drug release systems using different pH. In addition, flow cytometry and confocal fluorescent imaging proved CDs' cellular uptake and imaging performance. *In vitro* analyses further proved that the Chalcone-APBA-CDs exhibited a higher toxicity value than bare CDs and efficiently inhibited the proliferation of the HeLa cells depending on their dose-response. Finally, the performance of Chalcone-APBA-CDs on cancer healing capability was examined *in vivo* with fibrosarcoma cancer-bearing mice, which showed a remarkable ability to reduce the tumor volume compared with saline (control). This result strongly suggested that the Chalcone-APBA-CDs appear promising simultaneously as cancer cell imaging and drug delivery.

## Introduction

Cancer remained the world's deadliest disease, despite a worldwide investigation by many researchers 1. To date, chemotherapy, thermo-chemotherapy, and hyperthermia therapy have been the most commonly used techniques to kill cancer cells in a clinical setting. However, most of this therapy has many damaging side effects, which led to various other derivate diseases 2. Therefore, many efforts have been made to avoid the use of therapy by using advanced nanotechnology 3. Generally, fluorescent imaging and drug delivery systems using nanoparticles have been developed with various drug-free pharmacological properties and reduced toxic side effects 4. Recently, diverse materials functionalized CDs have been improved for monitoring intracellular bioimaging within divergent pH values 5. Therefore, these carbon dots (CDs) have been demonstrated to be highly promising drug carriers for bioimaging and delivery therapy owing to their excellent fluorescent behavior, tunable pore size, high surface area, and ability to penetrate deeply into cells 6.

On bioapplication, surface functionalization of CDs was essential to their successful compound delivery on the biological system. Diverse functional groups on the CDs allow covalent or non-covalent interactions with specific ligands and drugs to enhance their biological activity *in vitro* and *in vivo* 7. Furthermore, the CDs exhibiting sizes and dimensions are comparable to those of biomolecules targeted, such as DNA, proteins, and enzymes, which can facilitate an efficient binding to the specific receptor of proteins or DNA. However, the internalization of carbon dots into the cells depends on several mechanisms, such as receptor-mediated endocytosis, phagocytosis, and pinocytosis 8, 9. Recently, Wu et al. demonstrated the muti-functionalized carbon dots as an integrated solution for theranostic treatment using folate-polyethyleneimine passivated carbon dots (fc-rPEICdots). This nano design performed well on gene delivery in lung cancer therapy through the receptor-mediated endocytosis pathway 10. Since their antitumor effect and biocompatibility, CDs have been applied efficiently as specific targeting agents for diagnosing liver cancer and detecting the zinc ions concentration in the cells 7, 11, 12. The biocompatibility, low toxicity, photostability, and excellent photoluminescent properties of CDs prove this material to be a promising, safe live-cell staining agent and carrying drugs to target 13. Therefore, carbon dots (CDs) are interestingly used as efficient radiological contrast agents and fluorescent probes for X-ray CT imaging due to their excellent photoluminescence properties 14, 15.

The high optical properties of CDs play an essential role which has motivated many researchers to develop various techniques to increase such emissions 16, 17. Various reports have revealed that nitrogen doping onto CDs can effectively increase fluorescent properties 18. Nitrogen promoted additional orbital electrons that support increasing photon emission on CDs. According to Li et al., the N-doped carbon dots could be used for intracellular imaging in the HepG2 cells 19. On the other hand, studies on the application of Boron atom as a doping element upon CDs also reported the advantages of boronic acid-contained molecules to stimulate not only on optical properties of CDs but also the way of arraying the nanoparticle on microorganism targets 20, 21. Boronic acid can quickly conjugate with 1,2 and1,3-cis diols containing molecules such as sialic acid on the surface membrane of cancer cells through boronate ester formation 22. Hence, boronic acid becomes a targeting agent to improve the imaging accuracy and diagnosis of HeLa cells. Boronic acid receptors have also been utilized as a docking site for therapeutic agents and fluorescent dyes, according to the overexpression on the surface of various cell lines 23. Moreover, doping combination of Boron and Nitrogen on CDs has been reported to give multi-advantages to encourage the specifics of the nanomaterial to cancer cells 24-26. Wang et al. reported the synthesis of nitrogen and boron dual-doped graphene quantum dots as hydrophilic graphene quantum dots using 3-aminophenyl boronic acid, which could be applied as an imaging and photothermal therapy in the near-infrared second window system 27.

In the case of cancer therapy, some natural product molecules like chalcone have been widely used in biomedical fields owing to their antioxidant, anticancer, antiviral, and anti-inflammatory therapeutic activities 28. Organic chalcone molecules have introduced the ability to easily penetrate real-time staining into the organ, tissue, and tumorous cell 29. Furthermore, chalcone derivatives also display fluorescent activities, which can be used in various diseases by changing the emission color in living cells 30. Both electron donor and acceptor groups on the chalcone enhance the quantum yield and fluorescent intensity from inter-molecular change transfer supported by aprotic solvents 28. For instance, Zhou's groups reported the utilization of fluorescent chalcone as a chemical probe for investigating cellular targeting materials to determine their mechanism 31. As it naturally contains a hydroxyl (OH), chalcone and its synthetic analogs compound effectively exhibited antitumor activity, such as cell growth inhibition, cell proliferating inhibition, and chemo-preventing agents 32. This advantage came from the biomolecular interaction of chalcone with nucleic acids and amino groups. The substituent groups on chalcone may hinder significant side effects via the genotoxic process and decrease cancer cell cleavage 33. Moreover, Valdameri et al. investigated the methoxy effect on the chalcone structure, which inhibits the breast cancer resistance protein 34. Supporting previous reports, Bandgar and co-workers have shown that substituted groups (OCH3, OH, and halogen) contained chalcone emerge considerable anticancer activity (OCH3>OH>halogen) 33. Nevertheless, diverse substituent groups on the chalcone aromatic rings can be effectively active in the anticancer process 35.

Chalcone hybrids containing compounds have been employed as anticancer agents, and they possess efficient DNA binding agents which promote anticancer activity. The cell line inhibition activity of chalcone compounds using various tumor cell lines is shown in Table S1. However, using chalcone combined with N and B-doped CDs has not been intensively explored yet. Also, there is no report evaluating the release mechanism of chalcone molecules from CDs. The release model might include a crucial aspect of considering the clinical administration of natural products to treat cancer disease. Hence, the present work demonstrates the synthesis of chalcone-loaded carbon dots (Chalcone-APBA-CDs) for simultaneous cellular imaging and HeLa cancer cell therapy. Accelerating photoluminescence and cancer cell-specific targeting are organized by introducing APBA on CDs via *in situ* pyrolysis system. Besides the design of the synthesis condition, verification of the obtained Chalcone-APBA-CD is a special section discussed in this study, as important as chemical structure, fluorescent properties *in vitro*, and cytotoxicity analyses. For further investigation, the release model of chalcone optimized by varied pH, bioimaging capability, and *in vivo* analyses of Chalcone-APBA-CDs were pursued to prove the anticancer and bioimaging potencies of the CDs simultaneously.

## Experimental section

### Materials

CA (97%), 2-aminophenyl boronic acid (APBA, 97%), ethanol (EtOH, 97%), 3-(4, 5-dimethylthiazol-2-yl)-2, 5-diphenyltetrazolium bromide (MTT, 97.5%), sodium hydroxide (NaOH, 97%), and hydrochloric acid (HCl, 97%) were purchased from Sigma Aldrich (Milwaukee, WI, USA). In addition, 2**'**-hydroxy 2, 4-dimethoxy chalcone was obtained from the natural product laboratory. The Annexin V-FITC Apoptosis Detection Kit, Dulbecco's Modified Eagle's Medium (DMEM), normal saline (0.9%), phosphate-buffered saline (PBS), dimethyl sulfoxide (DMSO), and 4´,6-Diamidino-2-phenylindole (DAPI) were obtained from Merck, Germany. All chemicals were used as a chemical grade.

### Synthesis of fluorescent carbon dots (APBA-CDs)

As described in a previous study, the CDs were synthesized through a one-step pyrolysis process with some modifications 36. Firstly, 0.216 g of CA and 0.184 g of APBA were mixed and pyrolyzed at 250 ℃ for 4h in a muffle furnace. After the reaction, the CDs sample were cooled at 25 ℃ (room temperature). After that, the CDs were dissolved in 1mL sodium hydroxide solution (1M), and the distilled water was added to the CD solution until 5mL was obtained. Next, the resulting light-brown solution was neutralized using HCl (2M) and ultrasonicated for 30 min. After ultrasonication, the solution was centrifuged at 6000 rpm for 30 minutes to remove the larger particles and filter. Finally, the APBA-CD solution was collected for future use.

### Surface modification of CDs with Chalcone (Chalcone-APBA-CDs)

Surface-modified Chalcone-APBA-CDs were prepared by using the ultrasonication method. In brief, 10 mg of 2**'**-hydroxy 2, 4-dimethoxy chalcone was dissolved in 2 mL of ethanol solvent, and 3 mL of APBA-CD solution (5 mg/mL) and 20 mL of distilled water were added to the clear chalcone solution. The resulting solution was neutralized with hydrochloric acid (2 M). Next, a high-intensity ultrasonic probe (VCX 130 PB, sonics and Materials Inc., Newton, CT) was used to ultrasonicate the solution at 40% with 130 W power for 45 min. After the sonication process, the solution was centrifuged at 6000 rpm to remove large carbon particles and dialyzed using a cellulose dialysis bag (MWCO, 1000 Da) for 24 to remove free chalcone out. Finally, the clear sample solution was freeze-dried, and a pale-yellow powder was obtained for further characterization. The chalcone loading efficiency (LE) and the chalcone loading capacity (LC) values of the process were calculated using the below equations; 37


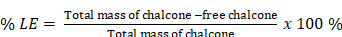

(1)


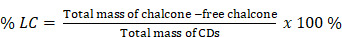

(2)

### *In vitro* cytotoxicity analysis

For MTT assay, 3-(4, 5-dimethylthiazol-2-yl)-2, 5-diphenyltetrazolium bromide was used to examine the cytotoxic activity of Chalcone-APBA-CDs on a HeLa cell line. The cells were cultured in a 96-well plate in DMEM medium with 10% FBS and incubated at 5% CO_2_ humidity at 37 ℃ for 24h. After 24h incubation, the incubated cells were washed twice with PBS (phosphate buffer saline, pH 7.4) to remove the medium. Subsequently, the cultured cell medium in each well was replaced by a serial concentration of Chalcone-APBA-CD solution and incubated at 37% for 24h. The wells were next washed with PBS solution to remove the cell medium. Then, a 300 μL medium containing MTT (DMEM 270 μL +MTT 30 μL) was added to each well, and the cells were further incubated for 4h at 37 ℃. At the end of the incubation period, each well's precipitate formed by the MTT result was dissolved using 200 mL of DMSO. The absorbance of each well was measured at 370 nm using GloMax-Multi Microplate Multimode Reader (Promega, USA)^38^. Identical cell culture procedures and conditions were used for the control experiments without the CD solution. The cell viability was calculated by the following equation: 39


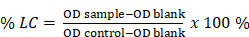

(3)

### *In vitro* drug release study

The kinetical release study of chalcone was carried out using the dialysis method. The release was determined by monitoring the chalcone concentration in Chalcone-APBA-CDs by passing the sample from the dialysis membrane bag (MWCO 1000). The drug release of Chalcone-APBA-CDs solution was determined under different pH conditions; one is in acid condition (pH-4), the second is a neutral condition (pH-7), and the last one is a primary condition (pH-9). The 10 mL of the Chalcone-APBA-CDs were taken in the dialysis membrane bag and immersed into a 100 mL PBS buffer solution. The entire system was kept at 37°C using magnetic stirring throughout the experiment. At specific time intervals, 3mL of the aqueous solution was taken from the release medium to measure drug release concentration using a UV-Visible spectrometer at 383nm 40. To control the total volume, recovered with the same volume (3mL) of fresh PBS. After that, the release concentrations were calculated using the following equation:

α = C_t_ + ν /v 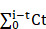

(4)

where α is the actual concentration at time t, 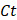 is the apparent concentration at time t, v is the total PBS volume, and 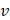 is the volume of the aqueous solution about taken. Four mathematical kinetic equations are employed for the evaluation of the dissolution profile. They are zero-order, first-order, Higuchi, and Korsmeyer-Peppas, which are explained below.

F_t_ = K_0_t (5)

ln _(1-Ft)_ = ‐K_1_t (6)

F_H_ = Khc t^1/2^ (7)

M_t_/M_α_ = K_kp_ t^n^ (8)

where 'F_t_' and 'F_H_' are the fractions of released chalcone in time t. M_t_ and M_α_ are the fractions of released chalcone in time t. 'K_0_', 'K_1_', 'K_hc_', and 'K_kp_' are model constants. 'n' is the diffusion exponent.

### *In vitro* analyses of Chalcone-APBA-CDs

As preparation, HeLa cells (human cervical cell lines, Institute of Tropical Disease Center) were seeded into 6-well plates and cultured in DMEM containing 1.5 gL^-1^ sodium bicarbonate, 10% fetal bovine serum (FBS), 1% L-glutamine, and 1% antimycotic antibiotic formulation at 37 °C inside the humidified incubation with 5% CO_2_. After incubation for 24 h, the carbon dots solution with 300 μL concentration at pH-7 was added to each well, and the cells were successfully one hour incubated. After that, the cells were washed with PBS three times to completely release unbound carbon dots and fixed with 7 % alcohol (10 min). After that, at room temperature, the cells were incubated with DAPI (0.05 μgml^-1^) in PBS for 21 min. After staining, the fluorescence image of cells was recorded under a laser scanning confocal microscope (Leica TCS SP2) with in-line Ar (488 nm) and He-Ne (503-608 and 588 nm) lasers.

### Tumor model and *in vivo* chemotherapy

Female mice (3-4 weeks of age) were implanted with fibrosarcoma cancer cells by subcutaneous injection. The mice were separated randomly into two groups as a control and treatment group (n = 8) for *in vivo* therapy experiments when the tumor volume appeared to be about 25-100 mm^3^. For *in vivo* chemotherapy study, the control group (Saline, 200 μL) and treatment group (Chalcone-APBA-CDs, 200 μL, 0.2 mg/mL^-1^) were intraperitoneally injected every two days for 31 days. After treatment, the tumor sizes were measured every other day by a vernier caliper ruler, and the tumor volume was calculated using the following equation: tumor volume= (length × width^2^) ⁄ 2. Also, the mice's weights were monitored every four days until 31 days. On day 31, after treatment, the tumor was removed and weighed at the end of the observation. In addition, about one mL of mice's blood was collected for the hematology analysis after the treatment of mice for 31 days.

### Characterizations

Fourier transforms infrared (FTIR) spectra were used to identify chemical bonds and functional groups with an IR Tracer-100 spectrometer (Shimadzu Inc., Japan) using the KBr pellet technique. The crystal structure properties of CDs were analyzed by X-ray diffraction (XRD, Rigaku D/Max-2BX, Japan), Cu Kα radiation (λ =1.54°A), and 18kW. Atomic force microscopy (AFM) images were measured using the AFM 5500M instrument (Hitachi Ltd., Japan). Thermogravimetric studies were performed from 40 to 1100°C with a rate of 10°C/min under a nitrogen atmosphere on a TGA 4000 (Perkin Elmer Inc., USA) instrument. The images of cells were demonstrated using Leica TCS SP2 inverted confocal microscope (Leica Microsystems Inc., Germany) instrument with a 63ⅹ 1.32 NA oil immersion objective, respectively. The nanomaterials' size diameter and surface zeta potential were determined with the Dynamic Light Scattering (DLS) instrument Delsa™ Nano HC Zeta Potential (Backman Coulter Inc., USA). The turbidity of the nanomaterial was determined by Hach 2100Q Portable Turbidimeter (Hach Inc., USA). UV-Vis absorption spectra were incubated with a Shimadzu 1800 spectrophotometer, and the photoluminescence (PL) spectra were acquired using PerkinElmer LS 55 spectrofluorometric (Perkin Elmer Inc., USA) equipped with a 20kW xenon lamp. With UV-Vis and PL data, the quantum yield percentage (%QY) of APBA-CDs and Chalcone-APBA-CDs were calculated using the following equation:



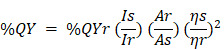



where I, A, and η correspond to the PL intensity, UV absorbance, and refractive index, respectively; along with Subscripts r and s represent the CDs sample and R6G dye, respectively. The PL decay lifetime of CDs was obtained using the time-correlated single photon counting (TCSPC) technique by Jobin-Yvon H10 monochromator with picosecond pulsed diode laser (PicoQuant PDL 200-B) at a wavelength of 450 nm.

### Statistical analysis

The result of CC50 (50% of cytotoxic concentration) and EC50 (50% of effective concentration) was carried out by using Origin software (version 8.0724, Origin lab Inc., Northampton, MA) with the dose-response non-linear fitting curve. Besides, *in vivo* experiments were performed, and investigated the means value by utilizing the student's t-test as statistical significance (*P < 0.05, **P < 0.01, and ***P < 0.001).

## Results and discussion

### Synthesis and characterization of Chalcone-APBA-CDs

**Scheme 1:**
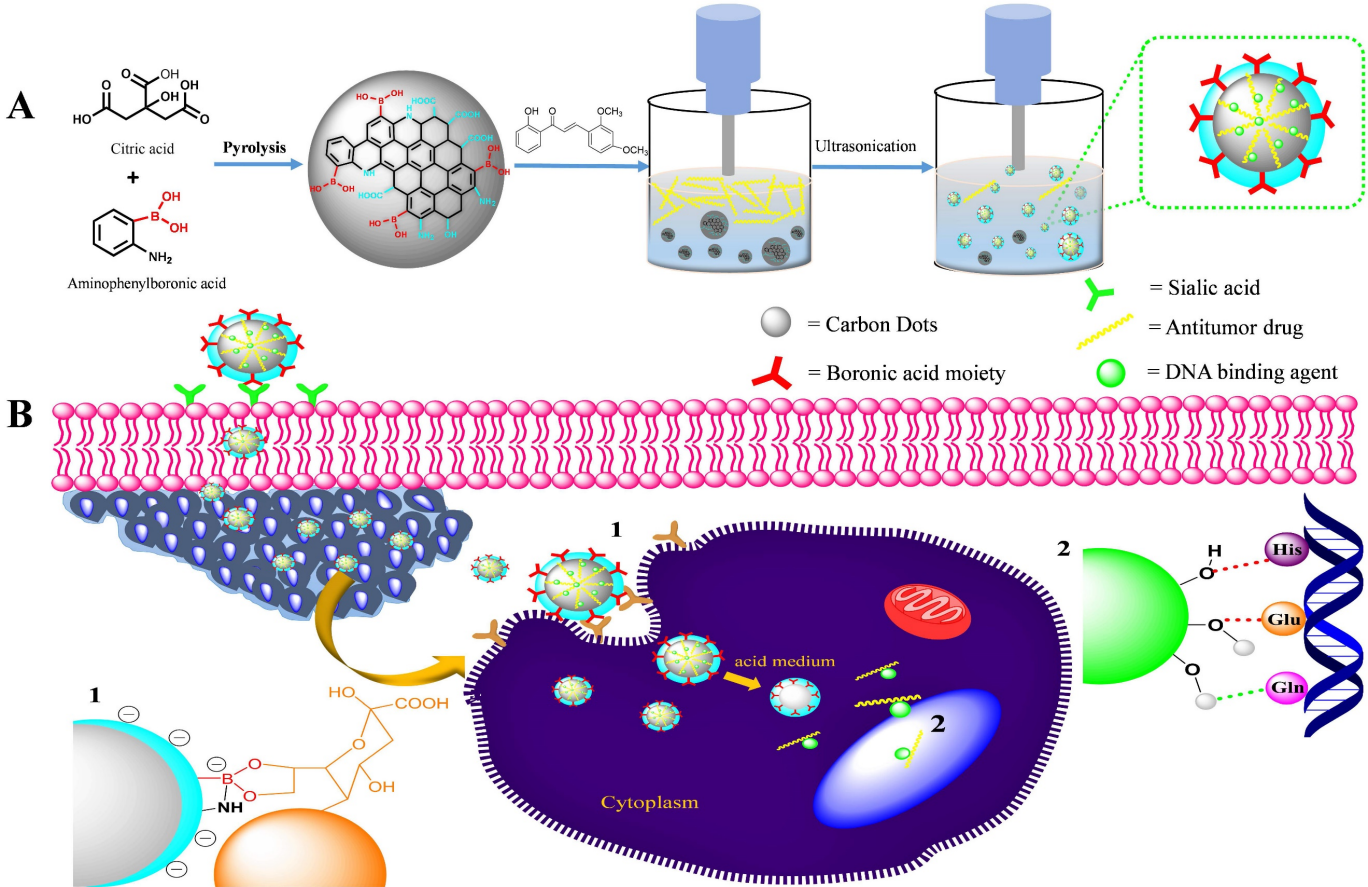
Schematic illustration for (A) synthesis of the Chalcone-APBA-CDs, and (B) fluorescent Chalcone-APBA-CDs as an intracellular staining agent on a cell membrane through H-bonding and electrostatic interactions

The CDs were synthesized via pyrolysis method mixing CA and 2-aminophenyl boronic acid (APBA), which has been reported HeLa cancer cell staining with highly photoluminescent activity 41. Furthermore, previous research has shown that boronic acid groups interact well with sialic acid on the tumor cell membrane 42. Then, the hydrophobic organic compound, 2'-hydroxy, 2, 4- dimethoxychalcone, was loaded on the CD surface after the pyrolysis reaction by ultrasonication of the physical interactions between the chalcone and CD surface (Scheme 1A). Therefore, we show that fluorescent Chalcone-APBA-CDs can be applied as an intercellular staining agent on a cell membrane through H-bonding and electrostatic interactions (Scheme 1B). Hence, it is beneficial to examine the biological activity of chalcone with electron donor and acceptor groups to investigate the cellular targets as chemical probes for cancer cells 28, 43.

**Figure 1:**
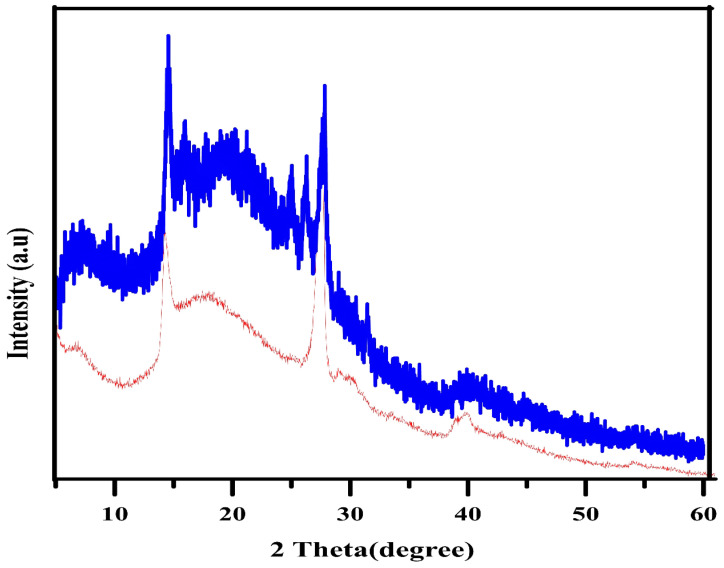
XRD patterns for as-prepared Chalcone-APBA-CDs (red) and APBA-CDs (blue).

**Figure 2:**
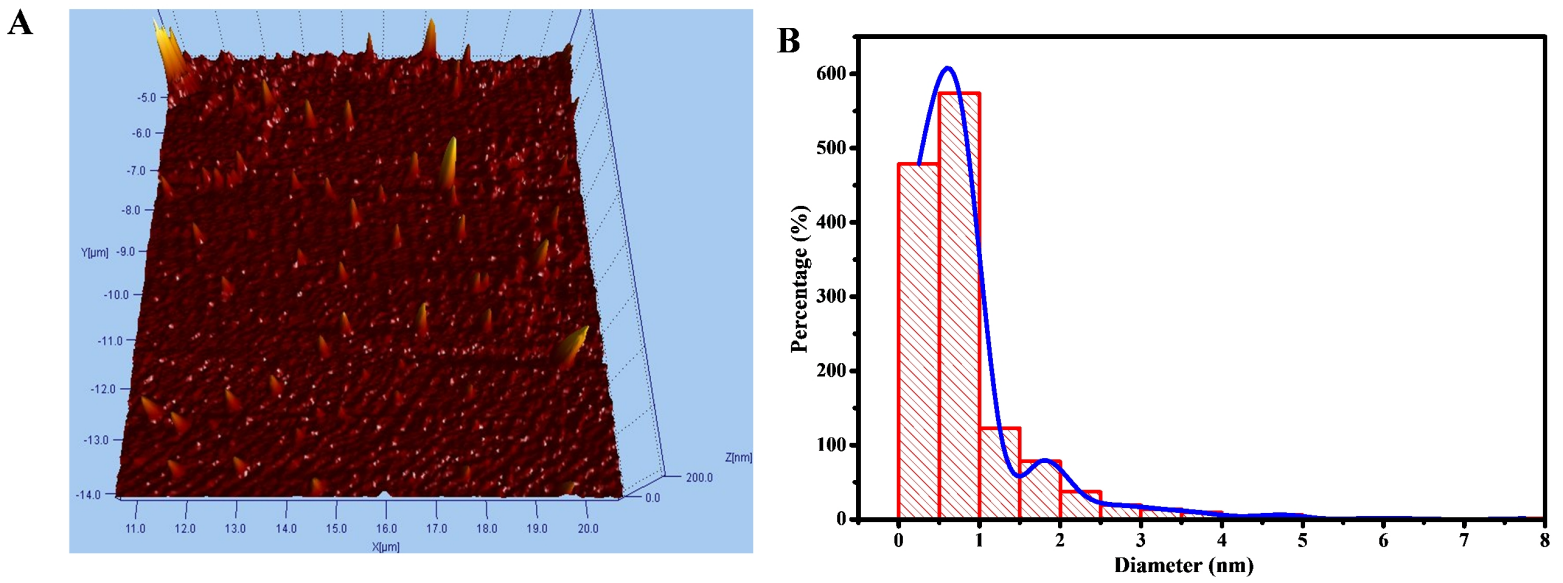
(a) AFM image of Chalcone-APBA-CDs, and (b) its histogram data.

The XRD patterns of both the chalcone-free CDs and chalcone-loaded CDs (Chalcone-APBA-CDs), shown in Figure 1., show a broad peak of chalcone-free CDs at 19.56 corresponds to the graphitic interplanar spacing of 0.45 nm. Several sharp peaks appear at around 27.8° (d=0.32 nm) and 14.51° (d =0.61nm), which shows the presence of boron and some highly crystalline carbon atoms on the CDs. Interestingly, the XRD spectrum of chalcone-loaded CDs displays similar peaks at 27.5° and 14.25°. However, the XRD pattern reveals a broad peak at 21.3° (0.42 nm), which suggests the presence of amorphous nature and related to Bragg's reflection of graphitic carbon 44. Further topography of Chalcone-APBA-CDs was observed by AFM (Figure 2), which revealed that the Chalcone-APBA-CDs show a spherical shape with an average size of approximately 0.5 nm. The above size diameter investigation was further validated with DLS analyses for comprehensive investigation (Figure S1a), which proved that colloidal modified CDs were below 10 nm. The analysis revealed that the size diameter of APBA-CDs is 7.531 nm and the introduction of chalcone slightly increases the diameter to 8.721 nm. Even if the increasing size diameter was insignificant, the colloidal formation of Chalcone-APBA-CDs performs relatively well against varied pH, ionic strength, and temperature (Figure S1b-d). Increasing turbidity value (up to 7 NTU) occurred on high pH as initial destruction of the colloidal system. Turbidity values of Chalcone-APBA-CDs ranged from 2-6 NTU, which is close to the control, indicating unaffected colloidal stability and prove well acceptable this material in biological circumstances of the human body.

**Figure 3:**
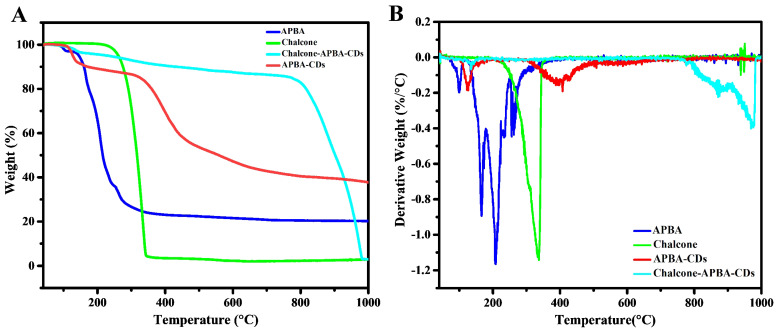
(a) TGA of APBA (blue), chalcone (green), APBA-CDs (red), and Chalcone-APBA-CDs (green-blue). (b) DTG analysis of APBA (blue), chalcone (green), APBA-CDs (red), and Chalcone-APBA-CDs (green-blue).

Thermogravimetric analysis and its differential thermogravimetric analysis (Figure 3) were further applied to determine the chemical bonding character of Chalcone-APBA-CDs. The data reveals the thermal stability of APBA, APBA-CDs, chalcone, and Chalcone-APBA-CDs, where weight loss of about 3.74% occurred on APBA starting from 60 ℃ to 130 ℃ referring to water molecules' volatilization. The degradation of bare APBA at 150-380 ℃ shows standard decomposition under endothermic conditions. APBA-CDs then demonstrate a slightly increased weight loss of 43.18% from 400 ℃ to 900 ℃, suggesting the functional group and materials decomposition and carbon degraded around 400 ℃-1000 ℃. The figure also shows decomposition at 250-350 ℃, with a weight loss of 96.3%, revealing the functional groups from the chalcone compound. Similar to the TGA curve of the Chalcone-APBA-CDs, it shows a weight loss of 13.91% from 120 ℃ to 770 ℃, suggesting the decomposition of the functional group-containing carbon particles. Chalcone-APBA-CDs show a higher weight loss (82.89%) between 770 ℃ and 975 ℃ than APBA-CDs, indicating destruction of the chalcone embedded. On the other hand, the last residual weight of Chalcone-APBA-CDs retains 3.2% compared to the actual amount of APBA-CDs (31.32%) at 1000 ℃. Interestingly, Chalcone-APBA-CDs have a similar residual weight (3.2%) at the same condition 45. The DTG spectrum of APBA, APBA-CDs, chalcone, and Chalcone-APBA-CDs shows the Chalcone-APBA-CDs own higher thermal stability at 882 ℃ and 975 ℃. This result may confirm a robust chemical structure of both chalcone and APBA-CDs, which chalcone compound can be loaded onto APBA-CDs 46.

**Figure 4:**
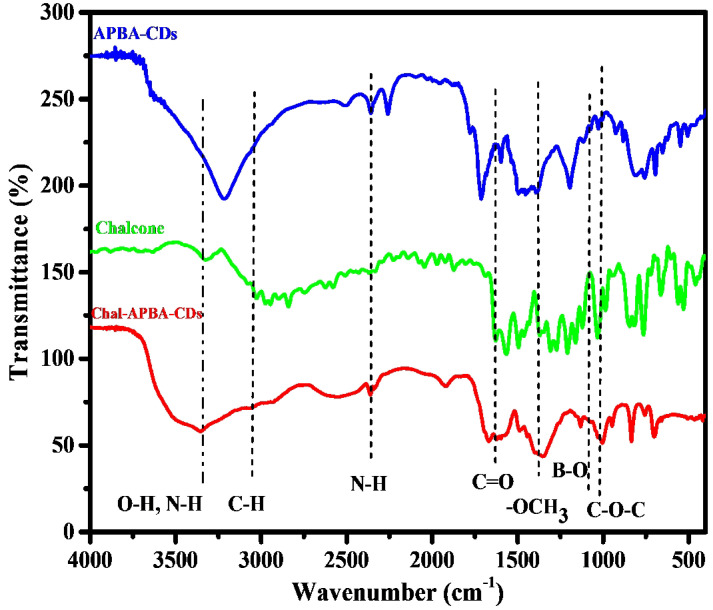
FTIR spectrum of APBA-CDs, chalcone, and Chalcone-APBA-CDs.

The incorporation of chalcone into the CDs also be revealed by zeta potential analysis; the APBA-CDs showed decreasing zeta potential value from - 15.5 mV to - 35.6 mV after the introduction of chalcone. The electrostatic character of the chalcone, which is dominant with carbon and oxygen elements, tends to have a negative charge on the chalcone and effect on decreasing of zeta potential value on Chalcone-APBA-CDs. In Figure 4, the Fourier Transform Infrared (FTIR) spectrum gives information about the functional group of APBA-CDs, chalcone, and Chalcone-APBA-CDs. The functional groups of Chalcone-APBA-CDs were compared with those of chalcone and APBA-CDs. The Chalcone conjugation was confirmed by an emerging band at 1628, 1596, 1368, and 1006 cm^-1^ and identified by the C=O, C=C, O-CH_3_, and C-O-C vibration bands 33, 43. The peaks at 1074, 1379, and 1031 cm^-1^ are correlated with C-B, B-O, and B-OH bonds. Strong broadband at around 3352 cm^-1^ is associated with O-H stretching, overlapping slightly with N-H stretching. Moreover, the absorption bands at 3352, 3062, 2926, and 2360 cm^-1^ are assigned to O-H and N-H, sp^2^ C-H, sp^3^ C-H, and N-H stretching vibration of Chalcone-APBA-CDs 47-50.

**Figure 5:**
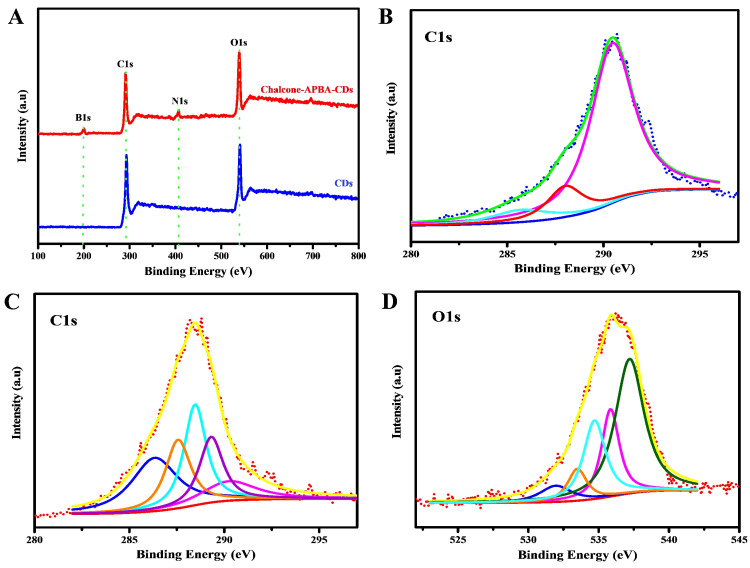
XPS scanning spectrum of (A) bare CDs and Chalcone-APBA-CDs. (B) High-resolution XPS survey scan of C1s for bare CDs. (C) and (D) High-resolution XPS survey scan of C1s and O1s for Chalcone-APBA-CDs.

The investigations to determine the graphitic structure and surface functional groups on CDs were further confirmed by X-ray photoelectron spectroscopy (Figure 5). The figure first confirms the elemental components of Chalcone-APBA-CDs consisting of carbon, oxygen, nitrogen, and boron (on 54.59%, 30.53%, 6.02%, and 8.39%, respectively). The high-resolution spectrum of C1s on Figure 5b shows five peaks on 286.3, 287.5, 288.4, 289.3, and 290.2 eV corresponding to graphitic carbon (C=C), hydroxy and methoxy carbon (C-OH, C-O-C), carbonyl (C=O), sp^2^ hybridized carbon with nitrogen and oxygen (N-C=O), and carboxylate (O-C=O) species, respectively. Further analysis of the O1s element (Figure 5c) shows five peaks for N-C=O, O-C (alcohol, ether), and B-O at 531.8, 533.6, and 534.6 eV, respectively 51. A satellite peak, a large number of sp^2^ carbon, and delocalized (π-π*) electrons showed at 535.9 and 537.2 eV 52. The above result showed that hydroxy, methoxy and boronic acid groups were integrally linked on the CD surface. In addition, the C1s analysis of bare CDs (Figure 5b) revealed three peaks at 285.6, 288.1, and 290.4 eV, which correlated to C-C, O-C=O, and C=O, respectively 53. Therefore, we concluded that the as-reported Chalcone-APBA-CDs exhibited a graphitic carbon nature and successfully facilitated multifunctional groups on the surface of CDs.

**Figure 6:**
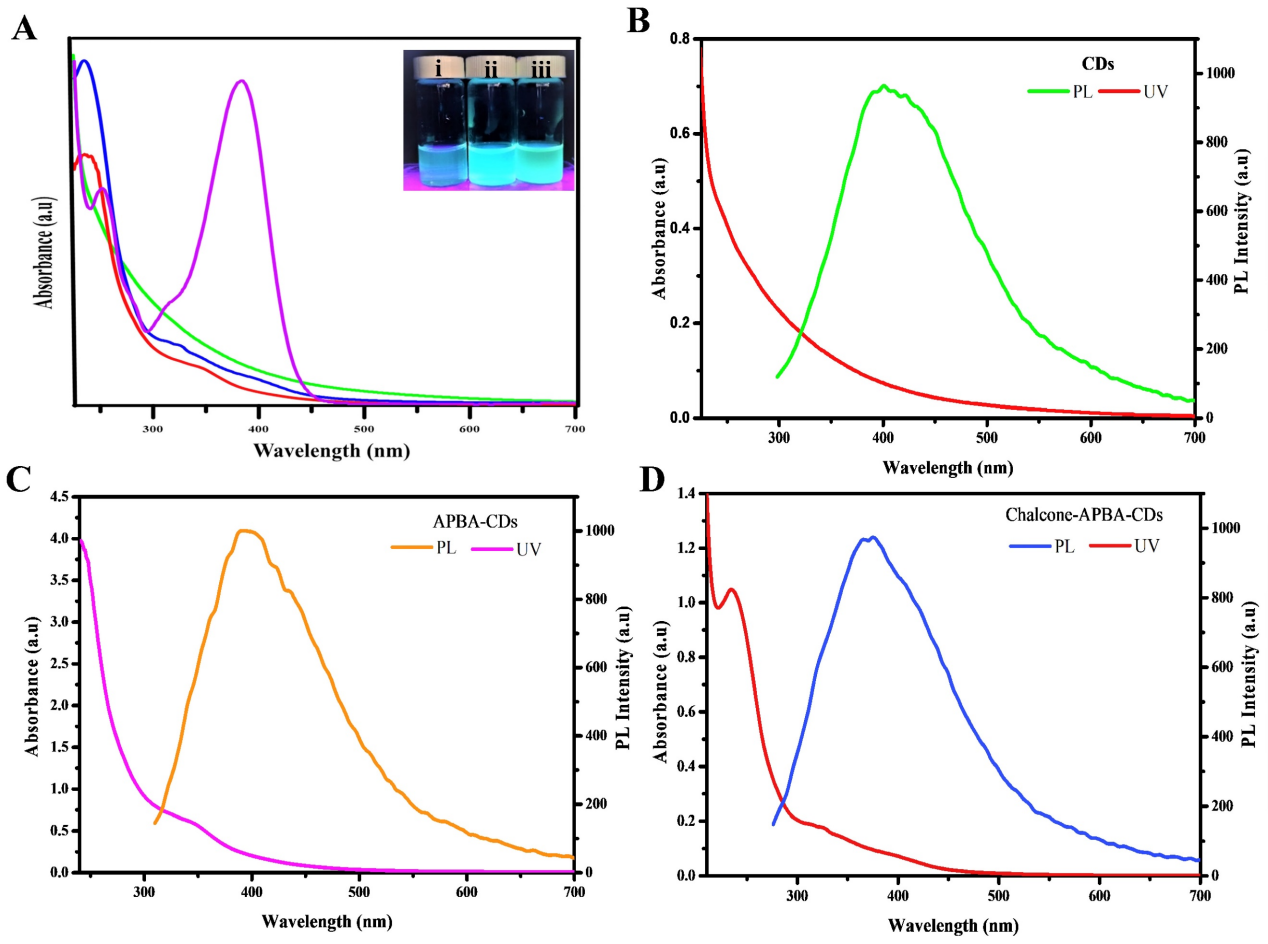
(a) UV-Vis absorption spectrum of CDs (green), chalcone (purple), APBA-CDs (red), and Chalcone-APBA-CDs (blue) with insert photograph of CDs (i), APBA-CDs (ii) Chalcone-APBA-CDs (iii) under 365 nm UV light (100 μg/mL). Comparison of the UV-Vis spectra with PL spectra of CDs (b), APBA-CDs (c), and Chalcone-APBA-CDs (d).

The absence of new binding from both bare CDs and Chalcone-APBA-CDs on XPS data also supports TGA on confirming that the attraction of chalcone to the CDs was served by physical interaction. UV-Vis and fluorescent spectrums were used to investigate further the optical properties of the CDs, chalcone, APBA-CDs, and Chalcone-APBA-CDs, as depicted in Figure 6. Figure 6a compares the absorption spectra of CDs, chalcone, APBA-CDs, and Chalcone-APBA-CDs. The UV-Vis absorption spectra of CDs show absorption bands at 245 nm and 305-358 nm, referring to the π-π* transition of aromatic carbon and n-π* transition. Chalcone solution has an absorption peak at 253 nm, which is related to the ℼ-ℼ* transition of C=C, while the broad absorption band at 314 and 384 nm can be described as n- ℼ* transition (C=O and C-O) on the chalcone compound. A comparison of the APBA-CDs and Chalcone-APBA-CDs showed absorption spectra below 262 nm and the broad shoulder peak at ~350 nm, correlated to the typical π-π* transition band of the C=C aromatic hybrid orbital and the n-π* transition band of the sp^3^ hybrid orbital.

Furthermore, the absorption band of Chalcone-APBA-CDs shows the spectrum at 235 nm, which is related to π-π* transition of sp^2^ hybridized carbon domains. Additionally, the absorption peak at 314 nm and the broad spectrum 388-423 nm are attributed to n-π* transition and surface moieties, respectively 54. Nevertheless, the absorption of Chalcone-APBA-CDs was slightly different from APBA-CDs and CDs due to their functionalized effect.

Further investigation on comparing the adsorption and photoluminescence properties of the CDs, APBA-CDs, and Chalcone-APBA-CDs are shown in Figure 6b-d. The CDs showed fluorescent emission peaks at 393 nm with 320 nm excitation wavelength. Moreover, the excitation wavelength of APBA-CDs and Chalcone-APBA-CDs showed the highest intensity peak at 385 nm and 388 nm, respectively. Figure S2 also confirms the fluorescent movement intensity of Chalcone-APBA-CDs to higher wavelengths on 320-520 nm with decreasing intensity 54. Thus, the Chalcone-APBA-CDs display excitation-dependent photoluminescence, which is related to the optical behavior of the various carbon sizes and surface defect activities of CDs 55. The optical analyses of the modified CDs further focused on their optical performance through the % QY value, where APBA CDs perform % QY up to 75%, then introducing chalcone to the CDs reduced the value to 17,6%. The decrease may happen due to absorption interference of the Chalcone compound. However, the QY value of Chalcone-APBA-QDs is still over that of bare CDs, which showed 12.4%. The data prove promising in utilizing CDs as detection agents for medical purposes. Improving on the QY investigation, the lifetime of both APBA-QDs and Chalcone-APBA-QDs were furnished on Figure S3 (supporting information), which showed slight PL lifetime values on 6.282 ns and 5.558 ns, respectively. The decreasing lifetime of Chalcone-APBA-QDs may be caused mainly by non-radiative decay on APBA QDs and enhanced area of the QDs after the introduction of chalcone.

### *In vitro* cytotoxicity

**Figure 7:**
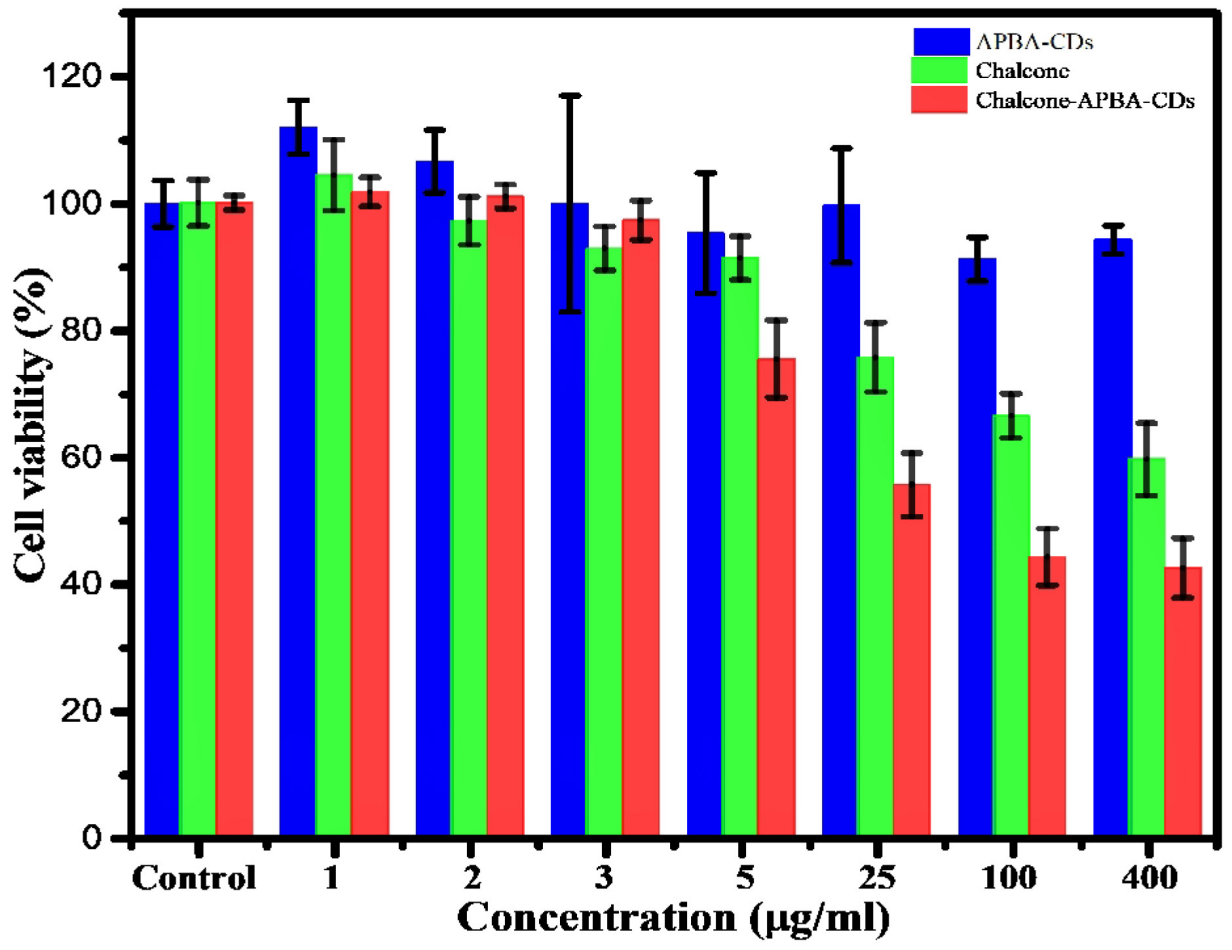
Cell viability data of HeLa cells incubated with APBA-CDs (blue), chalcone (green), and Chalcone-APBA-CDs (red). The data are demonstrated as means ±SD (n=3).

In order to investigate the cytotoxicity of Chalcone-APBA-CDs, we studied the viability of HeLa cells using an MTT assay. Figure 7 shows the cell viability of HeLa cells cultured with APBA-CDs, chalcone, and Chalcone-APBA-CDs for 24 h within the HeLa's viability was over 91% for APBA-CDs at a varied concentration (5-400 μg/mL). However, the toxicity of the chalcone compound was high with increased concentrations (25-400 μg/mL). Therefore, the cell viability of the chalcone effectively decreased. Compared with chalcone-loaded CDs, the viability of the HeLa cells dramatically reduced, from 74% to 42%, with a concentration range of 0.20-1.67 mg/mL due to the release rate of chalcone for 24 h from the CDs. In addition, Figure 7 also reveals that the cytotoxicity of Chalcone-APBA-CDs was significantly higher than the APBA-CDs and chalcone. It is proved by cytotoxicity concentration (CC_50_) of chalcone-free CDs, Chalcone, and chalcone-loaded CDs are 2.4, 4.5, and 5.4 μg/mL, respectively, as shown in Figure S4. Similarly, Yang et al. reported a comparison of doxorubicin-conjugated functional carbon dots (Dox-CDs) and Dox exhibited 70 % and 65 %, indicating that CDs may stimulate the work of the drug effectively 56. Thus, the chalcone-loaded CDs reduced the viability better than the chalcone-free CDs and revealed that Chalcone-APBA-CDs appear adequately for cancer cell imaging for diagnosis and therapeutic applications against cancer cells 57.

### *In vitro* drug release study

**Figure 8:**
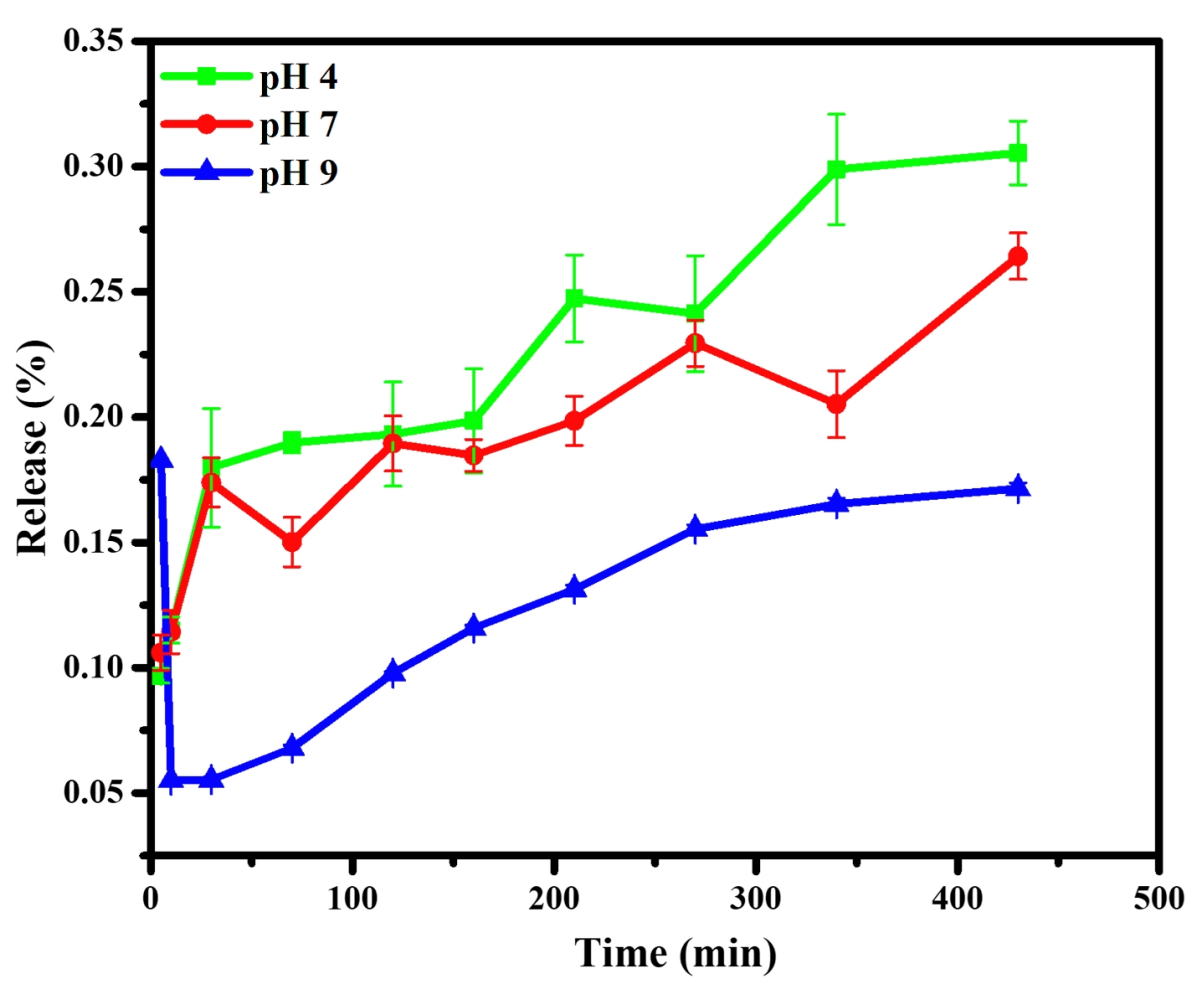
Drug release profiles of Chalcone-APBA-CDs at pH 4, 7, and 9. The data are demonstrated as means ±SD (n=3).

Further dissolution results for the chalcone upon CDs were monitored by its loading amount and loading efficiency, which reached 66.67% and 99.99%, respectively. This condition exhibits CDs as a suitable place for chalcone. In addition, the release profile of chalcone loading amount against varied pH showed on Figure 8. The existence of a benzene structure with abundant hydrogen atoms made the chalcone possess to interact with the CDs through hydrogen bonding, electrostatic and π-π stacking. However, the highest drug release profile shows in an acid medium due to electrostatic repulsion elaborated from chalcone and CD complexes in an acid condition. The chalcone's higher solubility in acid conditions is also responsible for the drug passing out and contributes on over release than others. This condition was strengthened by drug release study using Dox as reported in previous studies; in acidic intracellular regions, the Dox from CDs materials could be highly released due to the acid cleavage character of the Schiff base 5, 58. Therefore, the chemical structure similarity between chalcone and Dox made above assumption are acceptable. Moreover, compared to the acid condition, the attraction of chalcone is enhanced on more base conditions, through supporting on form hydrogen bonding and other physical attractions. These support previous data on FTIR and TGA, suggesting a chemical bonding interaction between the chalcone and CD surface.

### Study of Kinetical Release

**Figure 9:**
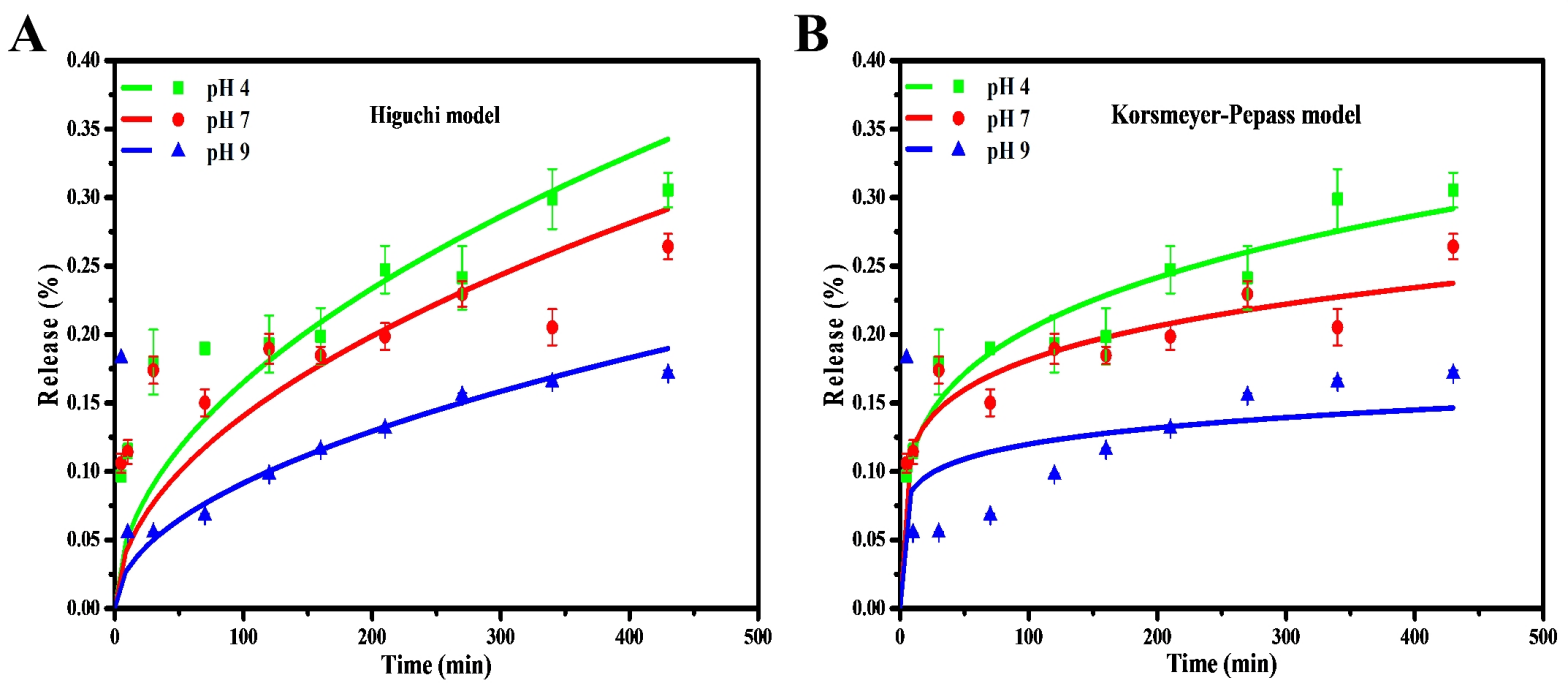
Drug release kinetic profile of Chalcone-APBA-CDs under varied pH conditions with (a) Higuchi, and (b) Korsmeyer-Peppas model. The data are demonstrated as means ±SD (n=3).

The kinetical release of chalcone from CDs was determined by using various kinetic models to analyze the kinetical character of the drug. The coefficient of correlation (R^2^) is related to the drug release kinetic models, which suggested that they were suitable models for a drug loading system. Herein, we fitted the drug-release data with different models, such as zero-order, first-order, Higuchi, and Korsmeyer-Peppas. The chalcone drug release showed almost zero dependence upon the pH at the initial stage and became relatively pH-dependent when over 10 mins. A continuous increase in the drug release was evident as the time increased up to 400mins in the Higuchi Model. The drug release system of chalcone is mainly suited to both Higuchi and Korsmeyer-Peppas models and displayed a higher correlation coefficient (R^2^) than zero-order and first-order models. This is a common phenomenon of releasing the drug from solid material 59. Compared to the Higuchi model, the chalcone diffusion was predicted to be closer to the Korsmeyer-Peppas model (higher R^2^ and lower chi-square), thus indicating that the polymeric-like structure of CDs affected the release process (Figure 9 and S5). Korsmeyer-Peppas model also performs the exponent of diffusion (n), which is correlated with drug release mechanisms (Table S2). The value is less than the diffusion exponent of the Fickian diffusion system, indicating that the release result relates to the spherical-shaped non-swelling particles. For the previous report on kinetic model calculation, zero-order and first-order models are not commonly followed because of fluctuating results 60. In addition, the chalcone drug release rate in the base condition (pH 9) is lower than in the acid and neutral conditions (pH 4 and 7). This release study demonstrated a time-dependent and pH-dependent release system from all kinetic models. Moreover, the proximity of the release pattern with Korsmeyer-Peppas was supported by lower Chi-square on all varied pH. The lower R^2^ and higher Chi-square values on pH 9 indicate that higher pH values damage the CDs and affect the release process 61.

### *In vitro* intracellular uptake and cell imaging

**Figure 10:**
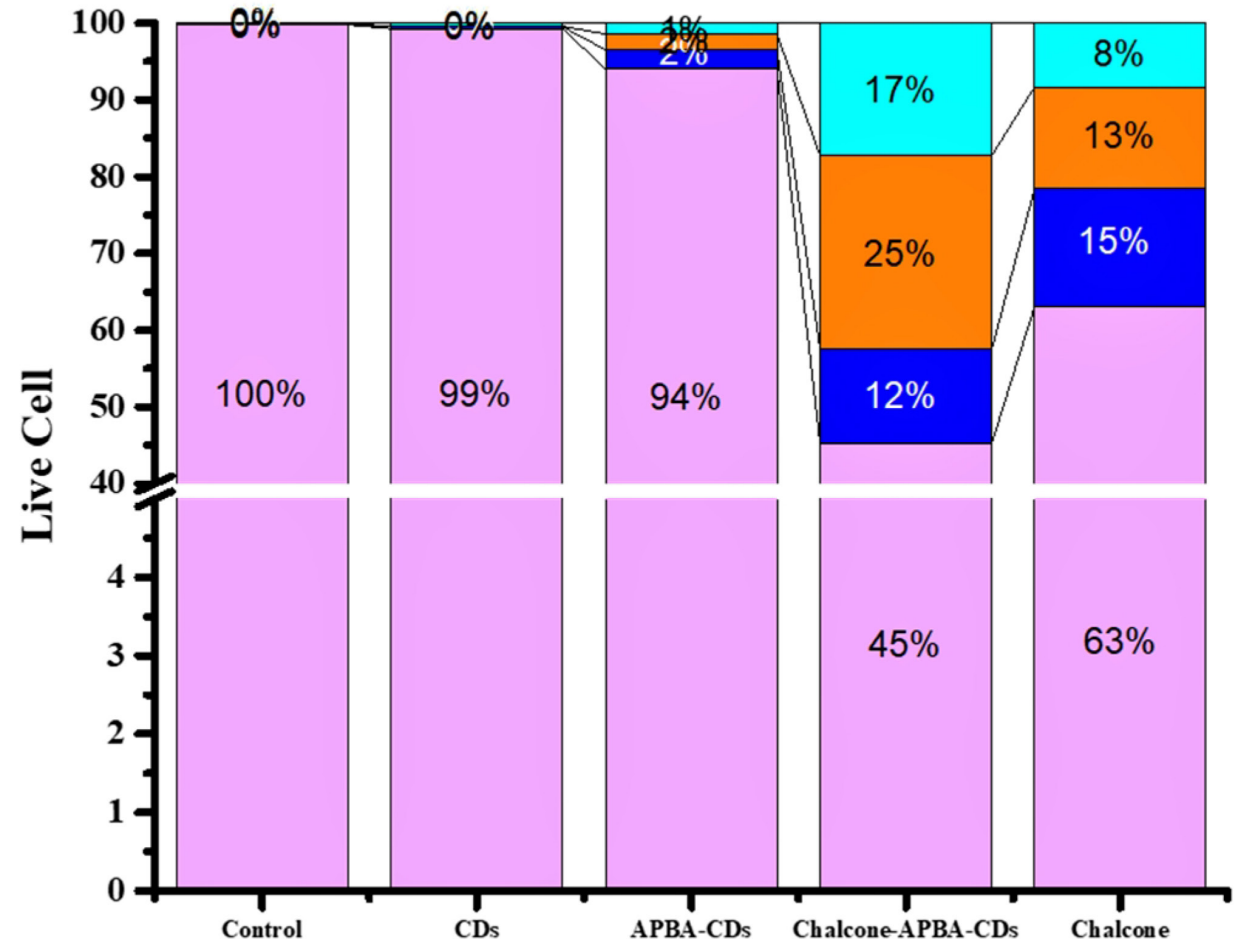
The flow cytometry data of CDs, APBA-CDs, chalcone, and Chalcone-APBA-CDs. Living cells (LT purple), early apoptosis (LT blue), late apoptosis (LT orange), and neurosis (LT green-blue).

The flowcytometry and confocal laser scanning microscopy (CLSM) was next performed to investigate the imaging capability and intracellular uptake of the Chalcone-APBA-CDs. Flow cytometry analysis can detect apoptotic and necrotic cells after the CDs treatment. The apoptosis of HeLa cells treated by CDs, chalcone-free CDs, chalcone, and chalcone-loaded CDs was analyzed by double-staining, using annexin V-FITC and propidium iodide (PI). Externalization of phosphatidylserine (PS) indicating apoptotic cells was determined by its conjugation with annexin V-FITC, whereas necrotic cells were identified by PI staining. As performed in Figure 10, there are no dead cells for control (without treatment, 100%). However, after incubated with CDs, APBA-CDs, and chalcone, the living cells were reduced to 99%, 94%, and 63%, respectively. This result indicates that CDs and APBA-CDs are low toxicity in HeLa cancer cells.

**Figure 11:**
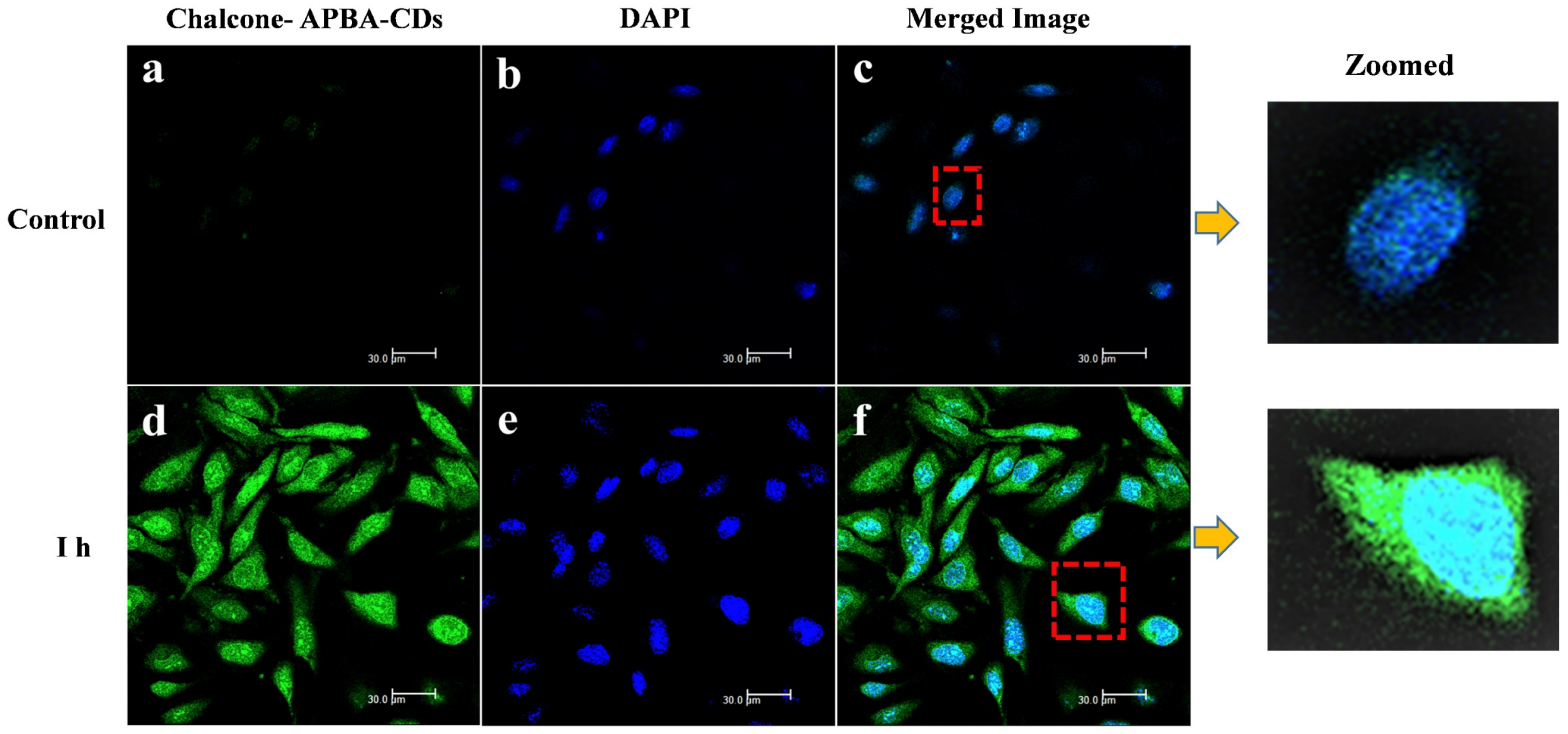
CLSM images of HeLa cells incubated with and without CD solutions and Chalcone-APBA-CDs solutions after incubation for 1 h (a and d). CLSM images of HeLa cells incubated with DAPI and Chalcone-APBA-CDs+DAPI (b and e). CLSM-merged images of HeLa cells incubated without CD and Chalcone-APBA-CDs solutions (c and f).

Moreover, the data reveal that the Chalcone-APBA-CDs displayed high toxicity for cancer cells with a significant percentage (45% for the normal cells); it was higher than nature chalcone (about 63%). This data also prove that a combination of chalcone performs better on cancer treatment. To get a deeper study on evaluating the optical properties of the CDs, Confocal Laser Scanning Microscopy (CLSM) is conducted to observe HeLa cancer cells treated with the CDs. As shown in Figure 11, the photograph of HeLa cells shows insignificant green fluorescence after one h treated with CDs. The cells perform the blue fluorescence referring to DAPI as a staining agent on the cell's nuclei.

**Figure 12:**
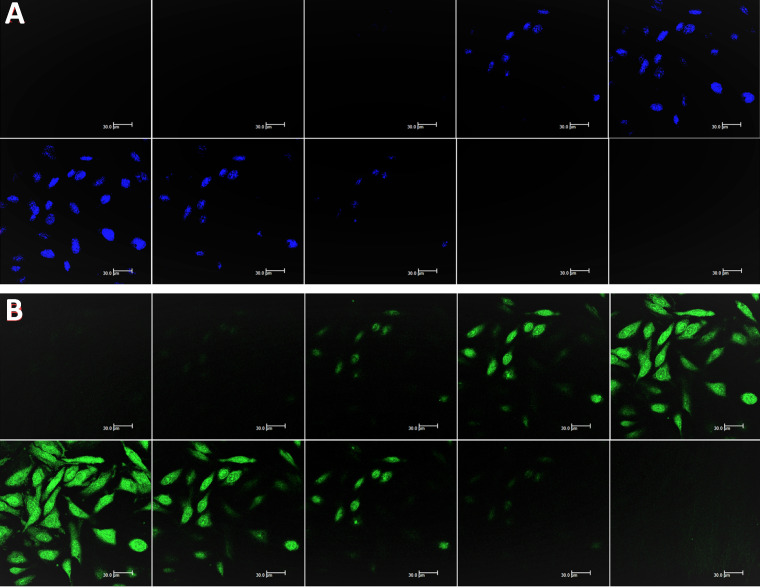
Z-stacking mode of CLSM (at 488 nm) on the HeLa cells images incubated with DAPI (a) and Chalcone-APBA-CDs, after 1h (b) from top (i) to bottom (x). The scale bar represents 30

Nevertheless, while the cell was treated with Chalcone-APBA-CDs, green emissions appeared to manifest successfully internalized the CDs onto the cytoplasm of the HeLa cell and DAPI on the nucleus area. This phenomenon also indicates that the boronic acid functional group on APBA can work properly to drive the CDs' internalization, specifically on targeted cells. This assumption is supported by our previous work investigating the insertion of boronic acid-attributed nanomaterial through the selective interaction of sialic acids at the surface of the cancer cells; and initiated endocytosis internalization 22. Furthermore, significant emission of Chalcone-APBA-CDs was explicitly found on the cytoplasm region of the cell, indicating endocytosis on insertion of the CDs. To further prove this point, the z-stacking mode on the CLSM image is furnished (Figure 12). The figure confirms that both DAPI and Chalcone-APBA-CDs pass cell membranes and exist on the cytoplasm area. This result reveals that Chalcone-APBA-CDs exhibit superior photostability and specific targeting drug delivery properties and are also beneficial for cancer cell imaging. Hence, this study's results show an excellent opportunity to overcome retroviral and cancer issues by designing CDs with a selective targeting agent toward specific receptors.

### Tumor model and *in vivo* chemotherapy

**Figure 13:**
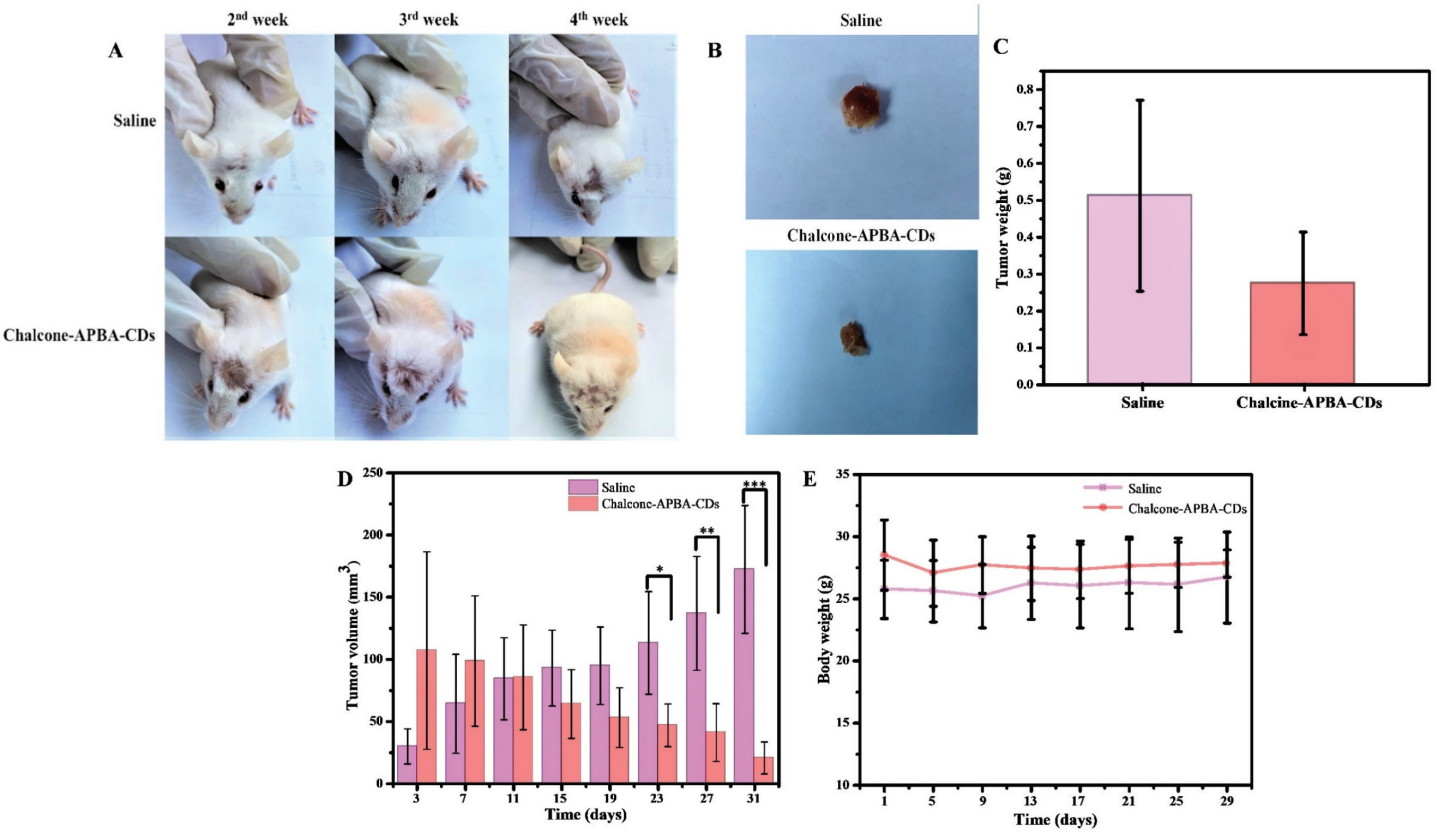
Photographs of (a) the syndromes of mice and (b) pictures of tumors after treatment with saline and Chalcone-APBA-CDs for 31 days. (c) Tumor weight, (d) tumor volume, and (e) body weight of treated mice within 31 days. The data are presented as means ± SD (n=8), *P< 0.05. **P< 0.01, ***P< 0.001.

Fibrosarcoma cancer-bearing mice were used further to investigate *in vivo* therapeutic efficacy of Chalcone-APBA-CDs to inhibit tumor growth activity. In this investigation, we first induced the mice with benzopyrene to grow the tumor (volume to be 25-100 mm^3^). Once the tumor showed, the mice were intraperitoneally treated by injection with saline (control) and Chalcone-APBA-CDs. As shown in Figure 13a, the physical apparition of tumor area of mice treated showed differences from initial treatment up to 4 weeks, in which mice treated with Chalcone-APBA-CDs showed size reduction of the tumor. This statement is validated by directly measuring and weighting the tumor size after mices are slaughtered (Figure 13b-c); up to 4 weeks, the mice's tumors decreased post-treated with Chalcone-APBA-CDs, and it was significantly smaller than the saline-treated groups. Improving the performance of the CDs, on Figure 2d, the differences on the tumor size were statistically significant when Chalcone-APBA-CDs were compared with the saline group (*P<0.05) 58. As typical on clinical cancer treatment, the antitumor drugs usually cause a reduction in the body weight of the mice 56. Thus, considering the treatment process's safety, the mice's weight is evaluated (Figure 13e). The data show that, up to the end of treatment, the average weight of the mice treated with Chalcone-APBA-CDs are not decreased significantly and is similar to the saline-treated as a control. These phenomena strongly indicated the non-toxicity feature highlighted by Chalcone-APBA-CDs and proved the clinical capability of Chalcone-APBA-CDs for the cancer treatment and well-delivering of the calcone to the target species.

**Figure 14:**
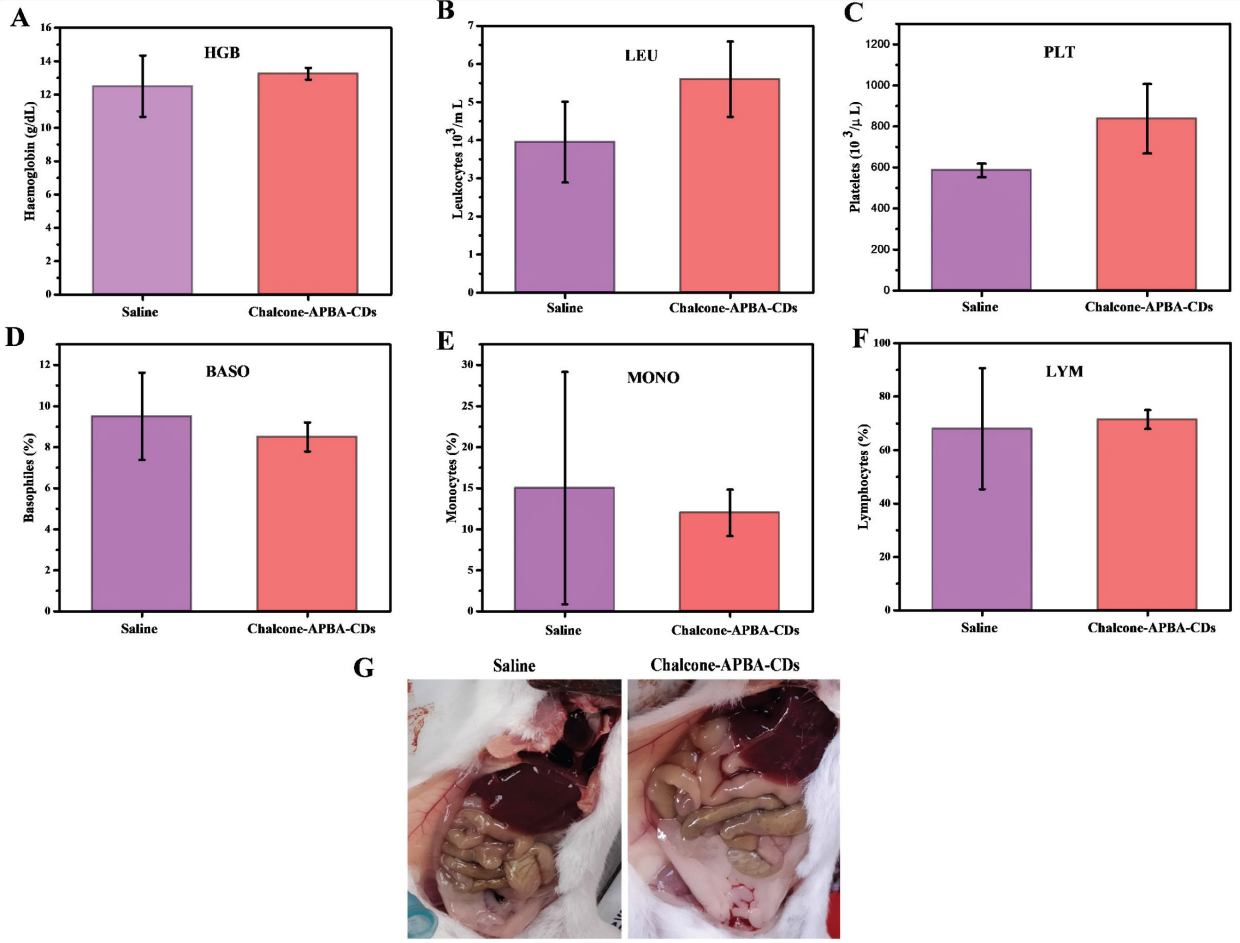
Hematology results of treated mice on 31 days, which show (a) hemoglobin (HGB), (b) leukocytes (LEU), (c) platelets (PLT), (d) basophiles (BASO), (e) monocytes (MONO), and (f) lymphocytes (LYM). (g) Pictures of mice's organs treated with saline and Chalcone-APBA-CDs (0.2 mg/kg, 200 μL).

Further improvement to evaluate any potential *in vivo* toxicity of saline and Chalcone-APBA-CDs on the treated mice, we further focus on hematology analysis. Figure 14 demonstrates the levels of important hematology markers, such as hemoglobin (HGB), leukocytes (LEU), platelets (PLT), basophils (BASO), monocytes (MONO), and lymphocytes (LYM). The result convinced no significant difference in blood counts between saline and Chalcone-APBA-CDs-treated mice. All the parameters above for HGB, LEU, PLT, and LYM remained within the normal reference range of mice, except the BASO and MONO counts. In this condition, the BASO and MONO results of Chalcone-APBA-CDs were higher than those in the normal range, and it followed the previous report claiming to save protocol on cancer treatment 63. The Chalcone-APBA-CDs also did not show noticeable adverse effects on organs such as the liver, kidney, and heart, as shown in (Figure 14g). From this observation, it is known that there were no color and morphological differences on both organs after 31 days treatment, supposing the CDs did not affect the organs except the cancer area and all the mice were alive till 31 days, referring to the nontoxic feature of Chalcone-APBA-CDs. In principle, when it has a toxic effect on the organs, the blood analysis may signal a significant difference from the control. Therefore, the hematology results suggested that the Chalcone-APBA-CDs nanoparticles showed no damage from the materials of CDs in the treated mice.

## Conclusion

In summary, we have synthesized the Chalcone-APBA-CDs combined with ultrasonic treatment. Chalcone is successfully loaded on the APBA-CDs, proven with several investigations. The *in vitro* assay reveals that Chalcone-APBA-CDs could reduce the HeLa cells' viability (55.4 - 42.3%) at a concentration of up to 400μg/mL. The loaded chalcone may release from the CDs and perform pH-dependent behavior with the highest drug release profile at acid medium for 430 min and follows both Higuchi and Korsmeyer-Peppas models. The Chalcone-APBA-CDs also sufficiently enter the cytoplasm through sialic acid receptor-endocytosis pathways and drive it to the DNA of the nucleus area. *In vivo* studies on applying Chalcone-APBA-CDs confirm a successful treatment process on mice by reducing the tumor size and having no damaging effect on the organs of mice post-treatment. These *in vivo* studies further support the *in vitro* analyses, confirming that CDs could efficiently work on clinical therapy by improving cancer imaging and enhancing anticancer activity with a selective targeting agent toward specific receptors in the tumor cell.

## Supplementary Material

Inhibition activity (IC50) of chalcone compounds using various tumor cell lines; UV-Vis absorption spectra of Chalcone-APBA-CDs and excitation-dependent PL emission spectra of Chalcone-APBA-CDs, inset: photograph images of water as a control and Chalcone-APBA-CDs under UV light at 365 nm; cell cytotoxicity data of HeLa cancer cells for 24 h incubation with APBA-CDs, chalcone, and Chalcone-APBA-CDs, the CC50 values were plotted on the red fitted curves, which determined from the dose-response on the Origin software (means ± SD (n = 3)); correlation coefficient of Chalcone-APBA-CDs at different pH media (pH-4, pH-7, and pH-9).

Supplementary figures and tables

## Abbreviations

CDs: carbon dots
Chalcone-APBA-CDs: chalcone loaded carbon dots
CQDs: carbon quantum dots
CA: citric acid
APBA: 2-aminophenyl boronic acid
EtOH: ethanol
NaOH: sodium hydroxide
HCL: hydrochloric acid
MTT: 3-(4, 5-dimethylthiazol-2-yl)-2, 5-diphenyltetrazolium bromide
DMEM: Dulbecco's Modified Eagle's Medium, PBS, phosphate-buffered saline
DMSO: dimethyl sulfoxide
DAPI: 4',6-Diamidino-2-phenylindole
Dox: Doxorubicin
FTIR: Fourier transform infrared, XRD, X-ray diffraction, AFM, atomic force microscopy
PL: photoluminescence
TGA: thermogravimetric analysis
CLSM: confocal laser scanning microscope
LE: chalcone loading efficiency, LC, chalcone loading capacity
CC50: 50% of cytotoxic concentration
EC50: 50% of effective concentration
HGB: hemoglobin
LEU: leukocytes
PLT: platelets
BASO: basophils
LYM: lymphocytes
MONO: monocytes
