# Supplementary material for: In vivo Study of Chalcone Loaded Carbon Dots for Enhancement of Anticancer and Bioimaging Potencies
Source: Nanotheranostics. 2023 Mar 12;7(3):281–98. doi: 10.7150/ntno.80030 (PMC10093417; doi:10.7150/ntno.80030)
Supplement: Supplementary file 1 — Supplementary figures and tables [file ntnov07p0281s1.pdf]

Supplementary material

In vivo Study of Chalcone Loaded Carbon Dots for Enhancement of Anticancer and Bioimaging Potencies

Mochamad Zakki Fahmi, [1, 2,*] Yu-Yu Aung,[1] Musbahu Adam Ahmad,[1] Alfinda Novi Kristanti,[1] Satya Candra Wibawa Sakti,[1*] Oka Pradipta Arjasa,[3] and Hwei Voon Lee [4]

1. Department of Chemistry, Airlangga University, Surabaya 60115, Indonesia.
2. Supramodification Nano-micro Engineering (SPANENG) Research Group, Airlangga University, Surabaya 60115, Indonesia.
3. Advanced Materials Research Centre - National Research and Innovation Agency, Central Jakarta 10340, Indonesia.
4. Nanotechnology & Catalysis Research Centre (NANOCAT), Level 3, Block A, Institute for Advanced Studies, Universiti Malaya, 50603 Kuala Lumpur, Malaysia.

Table S1. The inhibition activity (IC_{50}) of chalcone compounds using various tumor cell lines.

Compounds	Cancer cell lines	Cell line inhibition	Ref:
2,4-dimethoxychalcone	ACHN, Pancc 1, Calu 1, H460, HTC 116	90–95%	[1]
2'-hydroxy, 2-mehoxychalcone	CLBL-1	11.88 ± 1.58 µM	[2]
2'-hydroxy, 4-mehoxychalcone	CLBL-1	18.78 ± 2.3 µM	[2]
2'-hydroxy, 2,4-methoxychalcone	EAhy 926, HepG 2	10.73 , 31.26 µmol L^{-1}	[3]
2'-hydroxy, 2,4-methoxychalcone	HeLa cells	0.074 µM	[4]

*

* Corresponding author:

Department of Chemistry, Airlangga University, Surabaya 60115, Indonesia.

Supramodification Nano-Micro Engineering Research Group, Airlangga University, Surabaya 60115, Indonesia.

E-mail: m.zakki.fahmi@fst.unair.ac.id

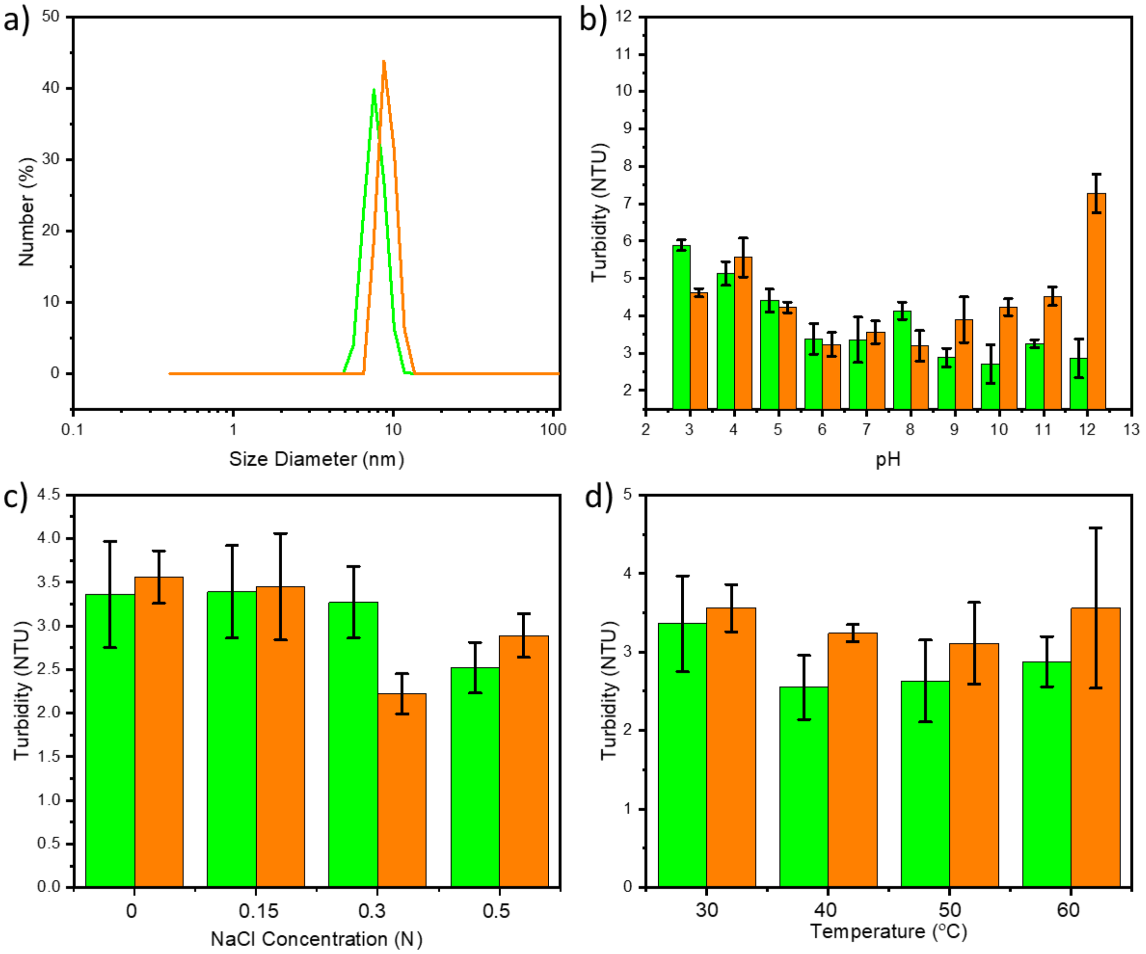

Figure S1. a) Size diameter of APBA-CDs (green) and Chalcone-APBA-CDs (orange) and its turbidity of on varied pH (b), NaCl Concentration (c), along with temperature (d); with n =3.

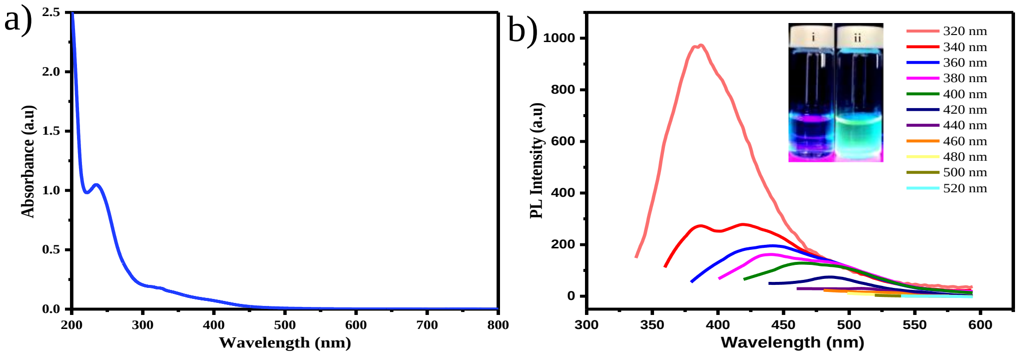

Figure S2. UV–Vis absorption spectra of Chalcone-APBA-CDs (a) and excitation-dependent PL emission spectra of Chalcone-APBA-CDs (b). Inset: Photograph images of water as a control (i), and Chalcone-APBA-CDs under UV light at 365 nm (ii).

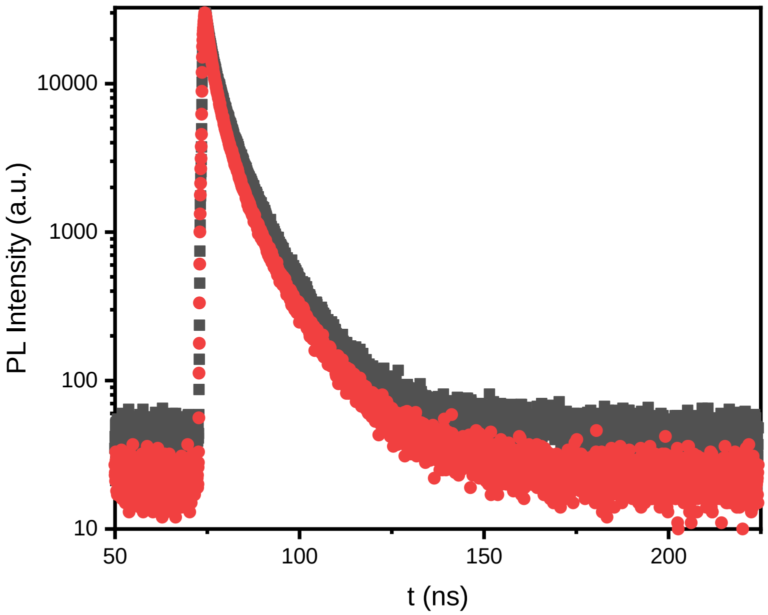

Figure S3. The fluorescence lifetime spectra of APBA-CDs and Chalcone-APBA-CDs observed with the excitation wavelength of 360 nm.

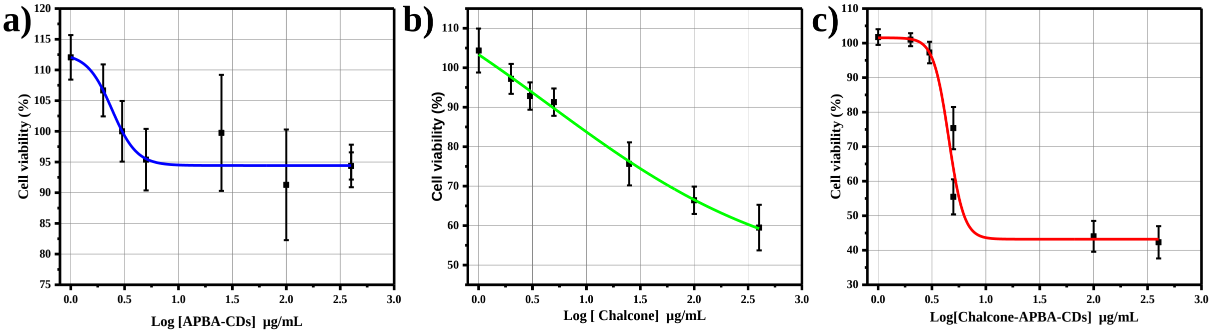

Figure S4. Cell cytotoxicity data of HeLa cancer cells for 24 h incubation (a) APBA-CDs, (b) chalcone, and (c) Chalcone-APBA-CDs. The CC50 values were plotted on the red fitted curves, determined from the dose response on Origin software. The data are shown as means ± SD (n = 3).

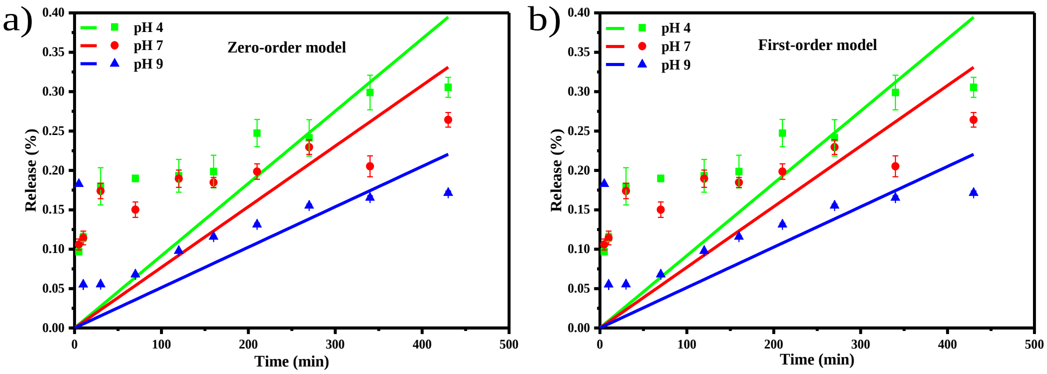

Figure S5. In vitro drug release kinetic models of Chalcone-APBA-CDs under pH 4, pH 7, and pH 9 conditions (a) zero-order and (b) first-order models.

Table S2. Correlation coefficient of Chalcone-APBA-CDs at different pH media (pH-4, pH-7, and pH-9).

APBA-CDs	Parameter	Zero-order	First-order	Higuchi	Korsmeyer-Peppas
**pH-4**	M_0 (g)	0.0032	0.0032	0.0032	0.0047
	K	9.17784E-06	9.19441E-06	0.0165	0.0652
	R^2	0.8639	0.8641	0.9269	0.9295
	Chi-square	4.7204	4.7101	0.3472	0.0246
	n	-	-	-	0.2471
**pH-7**	M_0 (g)	0.0032	0.0032	0.0032	0.0047
	K	1.06409E-05	7.71028E-06	0.0141	0.0802
	R^2	0.8178	0.8179	0.8709	0.8664
	Chi-square	3.8174	5.5019	0.4396	0.0153

	n	-	-	-	0.1766
**pH-9**	M_0 (g)	0.0032	0.0032	0.0032	0.0047
	K	5.12792E-06	2.6903E-06	0.0048	0.0326
	R^2	0.4037	0.4038	0.2968	0.1630
	Chi-square	13.3410	26.436	3.2638	0.9380
	n	-	-	-	0.1422

References

1. B. P. Bandgar, S.S. Gawande, R.G. Bodade, J.V. Totre, C. N. Khobragade, Synthesis and biological evaluation of simple methoxylated chalcones as anticancer, anti-inflammatory and antioxidant agents, Bioorg. Med. Chem. 18 (3) (2010) 1364-1370.
2. A. Pawlak, M. Henklewska, B. Hernández Suárez, M. Łużny, E. Kozłowska, B. Obmińska-Mrukowicz, T. Janeczko, Chalcone Methoxy Derivatives Exhibit Antiproliferative and Proapoptotic Activity on Canine Lymphoma and Leukemia Cells, Molecules. 25 (19) (2020) 4362.
3. X. Zarate, E. Schott, C.A. Escobar, R. Lopez-Castro, C. Echeverria, L. Alvarado-Soto, R. Ramirez-Tagle, Interaction of chalcones with ct-dna by spectrophotometric analysis and theoreticalsimulations, Quím. Nova. 39 (8) (2016) 914-918.
4. A. Kristanti, H. Suwito, N. S. Aminah, K. Haq, H.D. Hardiyanti, H. Anggraeni, N. Faiza, R. Anto, S. Muharromah, Synthesis of Some Chalcone Derivatives, in vitro and in Silico Toxicity Evaluation, https://rasayanjournal. co. in/admin/php/upload/917_pdf. pdf. 13 (1) (2020) 654-662.
